# Dietary Selenium Deficiency Exacerbates DSS-Induced Epithelial Injury and AOM/DSS-Induced Tumorigenesis

**DOI:** 10.1371/journal.pone.0067845

**Published:** 2013-07-04

**Authors:** Caitlyn W. Barrett, Kshipra Singh, Amy K. Motley, Mary K. Lintel, Elena Matafonova, Amber M. Bradley, Wei Ning, Shenika V. Poindexter, Bobak Parang, Vishruth K. Reddy, Rupesh Chaturvedi, Barbara M. Fingleton, Mary K. Washington, Keith T. Wilson, Sean S. Davies, Kristina E. Hill, Raymond F. Burk, Christopher S. Williams

**Affiliations:** 1 Department of Medicine, Division of Gastroenterology, Vanderbilt University School of Medicine, Nashville, Tennessee, United States of America; 2 Division of Clinical Pharmacology, Vanderbilt University School of Medicine, Nashville, Tennessee, United States of America; 3 Department of Cancer Biology, Vanderbilt University School of Medicine, Nashville, Tennessee, United States of America; 4 Department of Pharmacology, Vanderbilt University School of Medicine, Nashville, Tennessee, United States of America; 5 Department of Pathology, Microbiology, and Immunology, Vanderbilt University School of Medicine, Nashville, Tennessee, United States of America; 6 Vanderbilt Ingram Cancer Center, Vanderbilt University School of Medicine, Nashville, Tennessee, United States of America; 7 Veterans Affairs Tennessee Valley Health Care System, Nashville, Tennessee, United States of America; National Cancer Institute, United States of America

## Abstract

Selenium (Se) is an essential micronutrient that exerts its functions via selenoproteins. Little is known about the role of Se in inflammatory bowel disease (IBD). Epidemiological studies have inversely correlated nutritional Se status with IBD severity and colon cancer risk. Moreover, molecular studies have revealed that Se deficiency activates WNT signaling, a pathway essential to intestinal stem cell programs and pivotal to injury recovery processes in IBD that is also activated in inflammatory neoplastic transformation. In order to better understand the role of Se in epithelial injury and tumorigenesis resulting from inflammatory stimuli, we examined colonic phenotypes in Se-deficient or -sufficient mice in response to dextran sodium sulfate (DSS)-induced colitis, and azoxymethane (AOM) followed by cyclical administration of DSS, respectively. In response to DSS alone, Se-deficient mice demonstrated increased morbidity, weight loss, stool scores, and colonic injury with a concomitant increase in DNA damage and increases in inflammation-related cytokines. As there was an increase in DNA damage as well as expression of several EGF and TGF-β pathway genes in response to inflammatory injury, we sought to determine if tumorigenesis was altered in the setting of inflammatory carcinogenesis. Se-deficient mice subjected to AOM/DSS treatment to model colitis-associated cancer (CAC) had increased tumor number, though not size, as well as increased incidence of high grade dysplasia. This increase in tumor initiation was likely due to a general increase in colonic DNA damage, as increased 8-OHdG staining was seen in Se-deficient tumors and adjacent, non-tumor mucosa. Taken together, our results indicate that Se deficiency worsens experimental colitis and promotes tumor development and progression in inflammatory carcinogenesis.

## Introduction

The essential micronutrient selenium (Se) is of fundamental importance to human health. Se, through selenoproteins, has antioxidant roles, influences immune activity, and its levels are inversely correlated with cancer risk [1] and inflammatory bowel disease (IBD) [2]–[4] In the case of IBD, red blood cell glutathione peroxidase activity [5], a Se-dependent activity and mRNA levels of Sepp1 [6], a selenoprotein, are reduced.

IBD is an autoimmune inflammatory disease of the GI tract affecting as many as 1.4 million people in the United States [7]. IBD carries numerous and serious medical complications: stricture and fistula formation, infection, severe pain, anemia, and malnutrition [8]–[11]. The pathophysiology of IBD is thought to be multifactorial in origin. Genetic, microbial, and environmental factors have all been implicated, but the fundamental etiology remains unclear. Nutritional deficiencies have been a major concern in the management of IBD for decades and numerous studies have documented decreased levels of Se in IBD compared to control patients [12]–[15]. Moreover, as Se deficiencies are often seen in IBD, downregulation of several selenoproteins have also been identified. Serum selenoprotein P (Sepp1) levels are significantly lower in patients with Crohn’s disease [6] and glutathione peroxidase 1 mRNA is significantly downregulated in mononuclear cells from patients with IBD [16]. Defects in epithelial integrity contribute to IBD pathogenesis and understanding mechanisms affecting epithelial homeostasis is a priority [17]. In this regard, Se has been directly linked to WNT signaling [18] and the intrinsic apoptosis pathway [19], suggesting that it may play a role in epithelial integrity in response to injury.

Several epidemiological studies suggest that Se status is inversely correlated with cancer incidence and mortality [20]–[23], but Se supplementation trials failed to show benefit, possibly due to the fact that the advantages of Se supplementation are dependent on several factors including baseline Se status, genetic background (e.g. the existence of polymorphisms in selenoprotein genes), type of cancer, as well as the metabolic conversion and dose of Se supplements [24]. Furthermore, several selenoproteins have been correlated with cancer risk. Mutation of the selenocysteine transfer RNA gene which results in decreased expression of some selenoproteins results in reduction in murine colon cancer risk [25]. More specifically, elevated glutathione peroxidase-2 (Gpx-2) expression has been detected in colorectal adenomas [26]–[29]. Subsequently, double knockout of Gpx-1 and Gpx-2 leads to bacterial-induced intestinal inflammation and, ultimately, ileal tumor formation [30] and Gpx-2 decreases inflammation and tumors in response to inflammatory carcinogenesis [31]. Expression levels of selenoproteins including Gpx-1, Gpx-3, and Sepp1 are significantly decreased in colorectal cancer, with lowered Sepp1 expression correlating most significantly with tumor stage [32]. As such, we have shown that Gpx-3 serves as a tumor suppressor in the AOM/DSS model of CAC [33]. Thus, both selenium and selenoproteins may play pivotal roles in tumor modulation, especially in the setting of inflammatory carcinogenesis.

There may be sub-populations of CRC patients who would benefit from Se supplementation. IBD may fall into this category as it is characterized by chronic inflammation and oxidative stress [34] and ulcerative colitis and Crohn’s colitis patients are at increased risk for developing colon cancer [35]. Experimental studies demonstrating that Se deficiency exacerbates intestinal injury and tumorigenesis are not definitive. The aim of these studies was to determine whether Se deficiency exacerbates colitis and promotes inflammation-associated carcinogenesis. Mice were rendered Se-deficient via maintenance on a tightly controlled Se-deficient diet. Se-deficient mice had exacerbated colitis after DSS injury, resulting in a pro-tumorigenic microenvironment with increased cytokines, oxidative stress, and DNA damage. Furthermore, when inflammatory carcinogenesis modeling was performed, Se-deficient mice had increased tumorigenicity and more advanced lesions compared to Se-sufficient cohorts. Thus, Se supplementation in Se-deficient IBD patients may protect from mucosal injury and malignant degeneration.

## Materials and Methods

### Ethics Statement

This study was performed in strict accordance with the recommendations in the Guide for the Care and Use of Laboratory Animals of the National Institutes of Health. The protocol was approved by the Institute of Animal Care and Use Committee at Vanderbilt University (protocol number: M/10-355). Every effort was made to minimize suffering.

### Murine DSS Morbidity and Inflammatory Carcinogenesis Modeling

Experimental diets were Torula yeast-based and identical except for their Se contents. The basal diet contained less than 0.01 mg Se per kg (Se-deficient) and the Se-sufficient diet was the same diet supplemented with 0.25 mg Se as sodium selenite per kg. Se-deficient and -sufficient diets were prepared and pelleted to our specifications [36] by Harlan-Teklad (Madison, WI, USA). Upon weaning, Wild-type (WT) C57BL/6 mice were maintained on the experimental diets for 12 weeks prior to initiation of DSS or AOM/DSS experiments to ensure Se deficiency.

DSS at a molecular weight of 40,000–50,000 was obtained from USB Corporation (Cleveland, OH, USA). A single lot was used for all experiments described in this publication. A 3% w/v DSS solution was made in distilled water and filtered through a 0.22 µm cellulose acetate filter. This solution was then substituted for animal drinking water and barriers were placed on the automatic watering systems within each cage.

**Figure 1:**
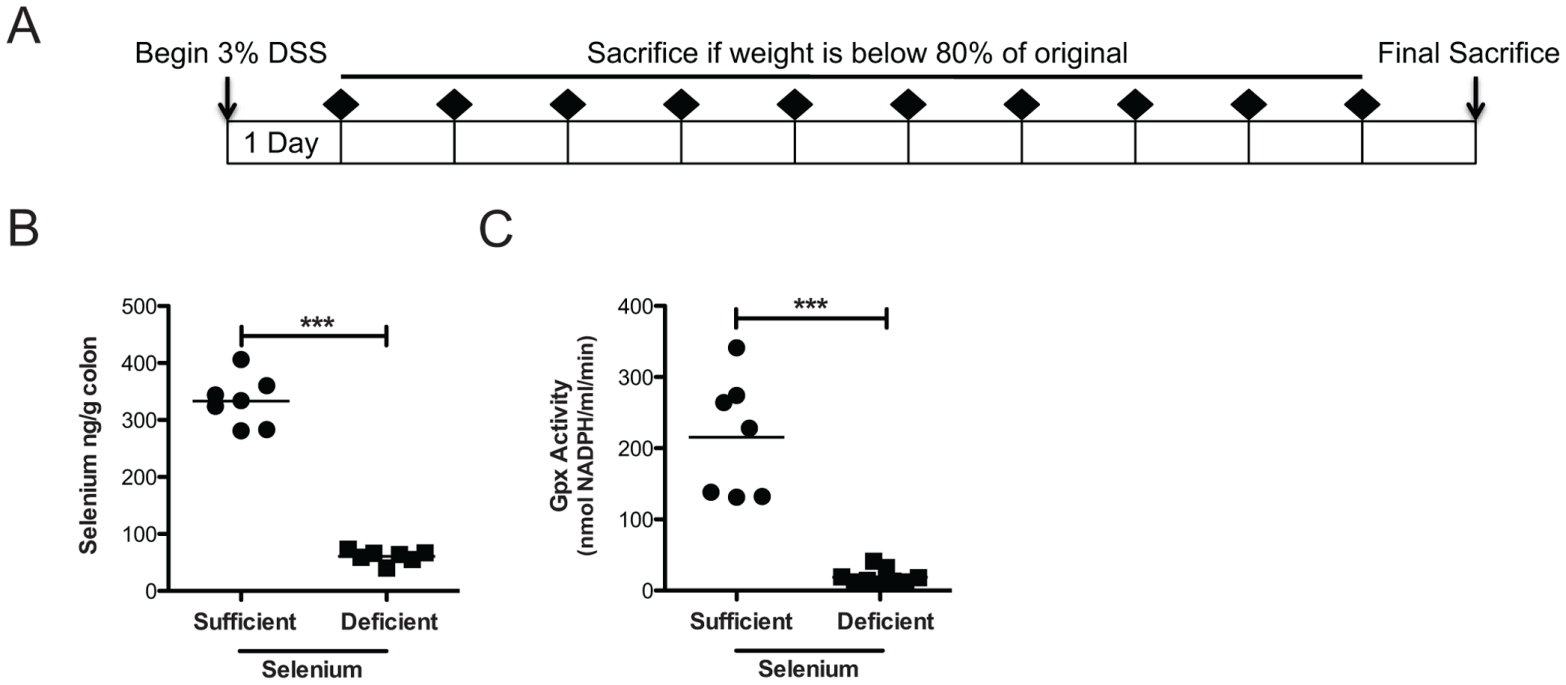
Dietary selenium depletion reduces colon selenium levels and plasma glutathione peroxidase activity. A. Schematic for the DSS morbidity protocol. At day 0, mice were weighed and then given a 3% DSS solution in lieu of drinking water. Each day, mice were weighed and stools were obtained for analysis of blood and stool consistency (weight and stool analyses times indicated by black diamonds). Individual mice were sacrificed if their weights fell below 80% of their original weights. B. Selenium sufficient (n = 7) and deficient (n = 7) colons were analyzed for selenium concentrations. ***P<0.0001. C. Plasma glutathione peroxidase activity is quantified. ***P<0.0001.

**Figure 2:**
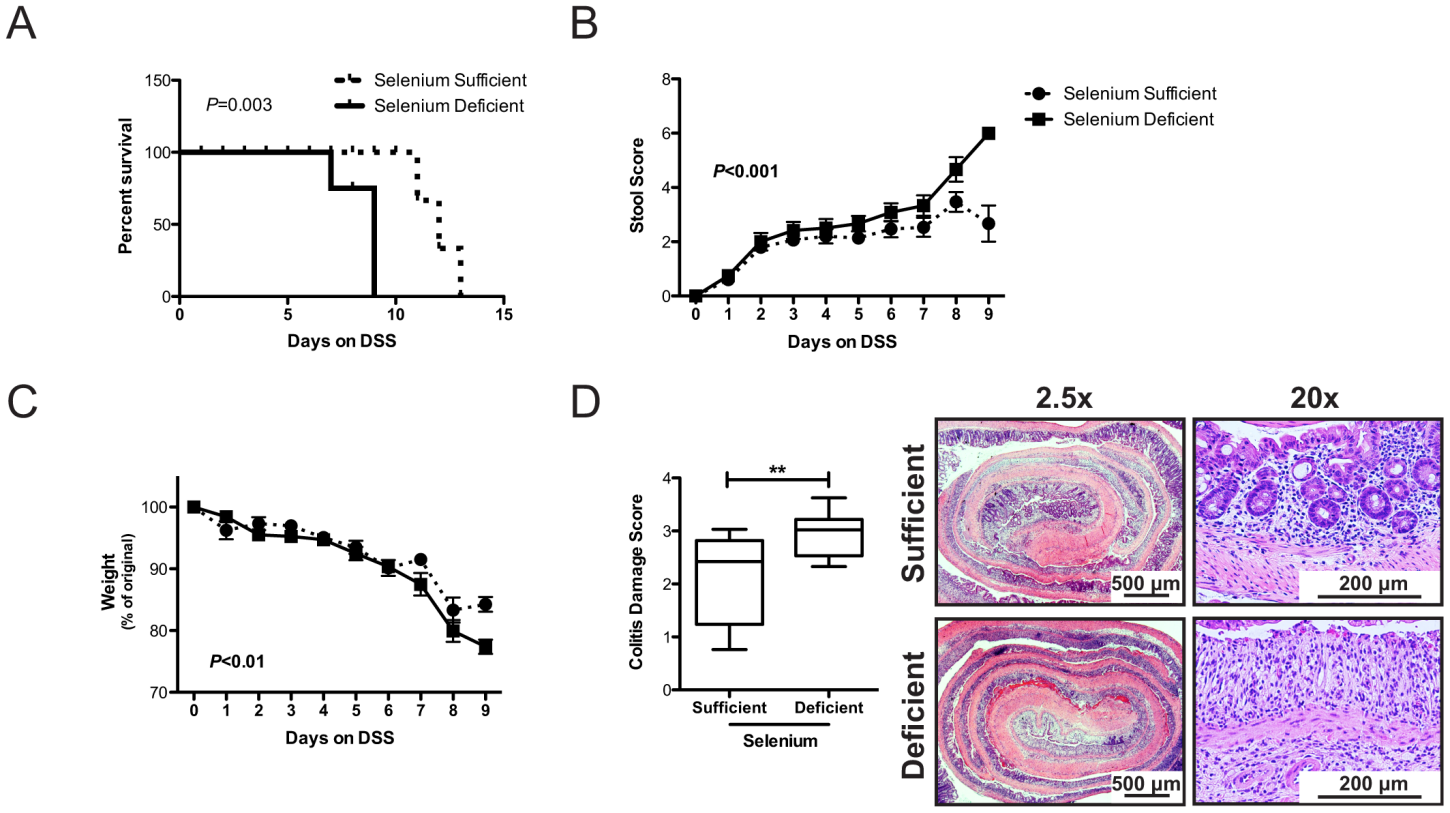
Selenium deficiency exacerbates colonic injury after DSS treatment. A. Se-sufficient (n = 15) and -deficient (n = 10) mice were subjected to the DSS morbidity protocol (see Figure 1A) and percent survival was tracked. *P* = 0.003, Log-rank (Mantel-Cox) test. B. Stool scores were analyzed for Se-sufficient and -deficient mice on the DSS morbidity protocol. The graph depicts the average stool score for each dietary group on each day of DSS administration. *P*<0.001, 2-way ANOVA. C. Percent of original weight averaged for each dietary group on each day of DSS administration. *P*<0.01, 2-way ANOVA. D. Colitis damage scores (described in Materials and Methods) for each dietary group analyzed at the completion of the experiment (left) and representative images of Swiss rolled colons from each group (right). Top and bottom of box 25^th^ and 75^th^ percentile, bar represents the median value, whiskers minimum and maximum values. **P = 0.006, Students-t test.

**Figure 3:**
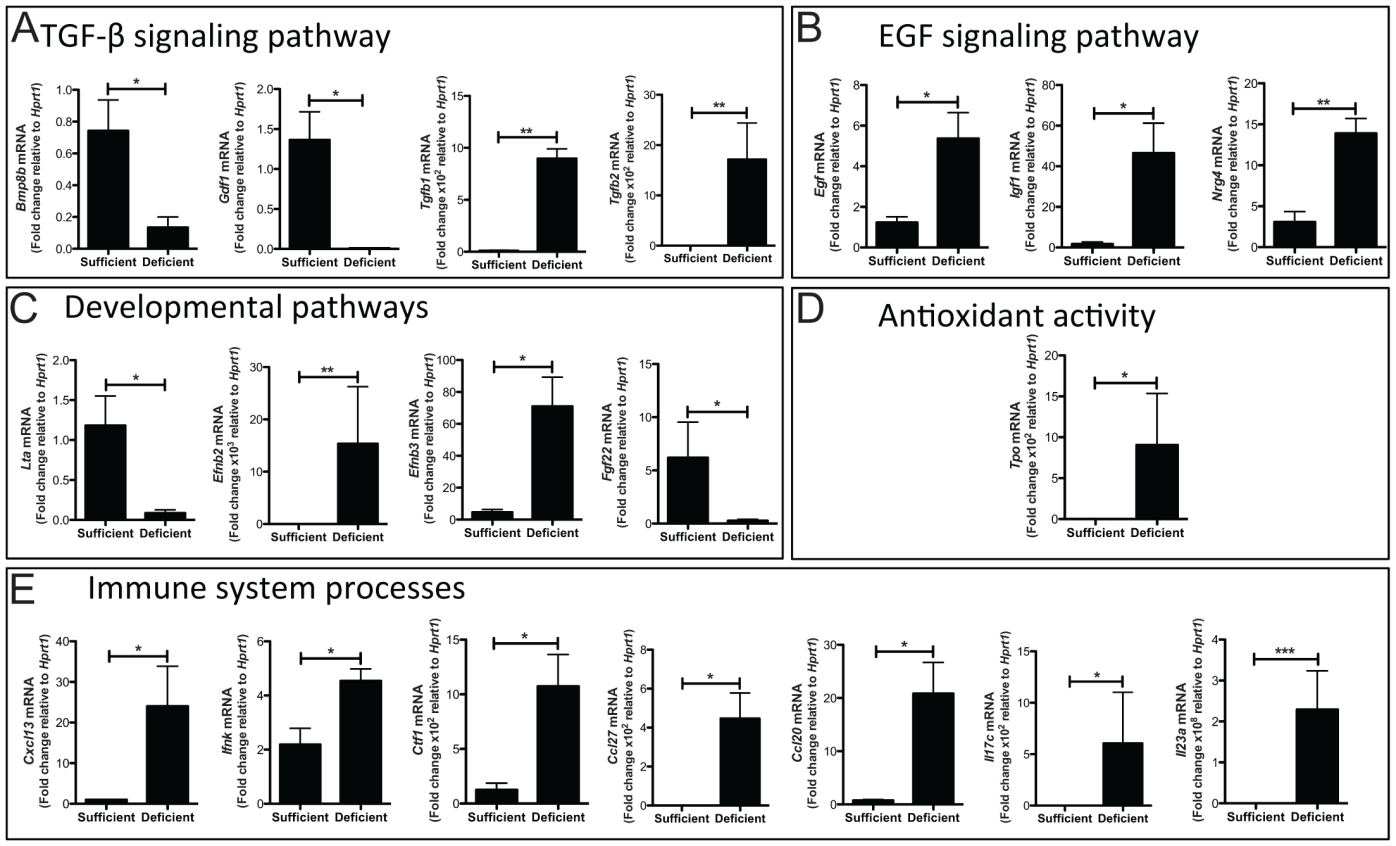
Cytokine expression is aberrantly regulated in selenium deficient mice post-DSS. A. mRNA expression changes in Se-sufficient (n = 4) and -deficient (n = 4) mice on from day nine of the DSS morbidity protocol. Genes were grouped based on pathways identified by PANTHER pathway analysis software. Expression was normalized to *Hprt*. A. TGF-β pathway alterations include Bmp8b = bone morphogenetic protein 8b, Gdf1 = Growth differentiation factor-1, Tgfβ1 = transforming growth factor beta-1, Tgfβ2 = transforming growth factor beta-2. B. EGF pathway alterations include Egf = epidermal growth factor, Igf1 = insulin-like growth factor 1, Nrg4 = neuregulin 4. C. Developmental pathway alterations include expression changes in Lta = lymphotoxin alpha, Efnb2 = ephrin-B2, Efnb3 = ephrin-B3, Fgf22 = fibroblast growth factor 22. D. The gene altered within the group titled antioxidant activity is Tpo = thyroid peroxidase. E. Immune system process alterations are demonstrated in Cxcl13 = chemokine (C-X-C motif) ligand 13, Ifnk = interferon kappa, Ctf1 = cardiotrophin 1, Ccl27 = chemokine (C-C motif) ligand 27, Ccl20 = chemokine (C-C motif) ligand 20, IL17c = interleukin 17c, IL23a = interleukin 23a.­­ *P<0.05, **P<0.01, ***P<0.001.

**Figure 4:**
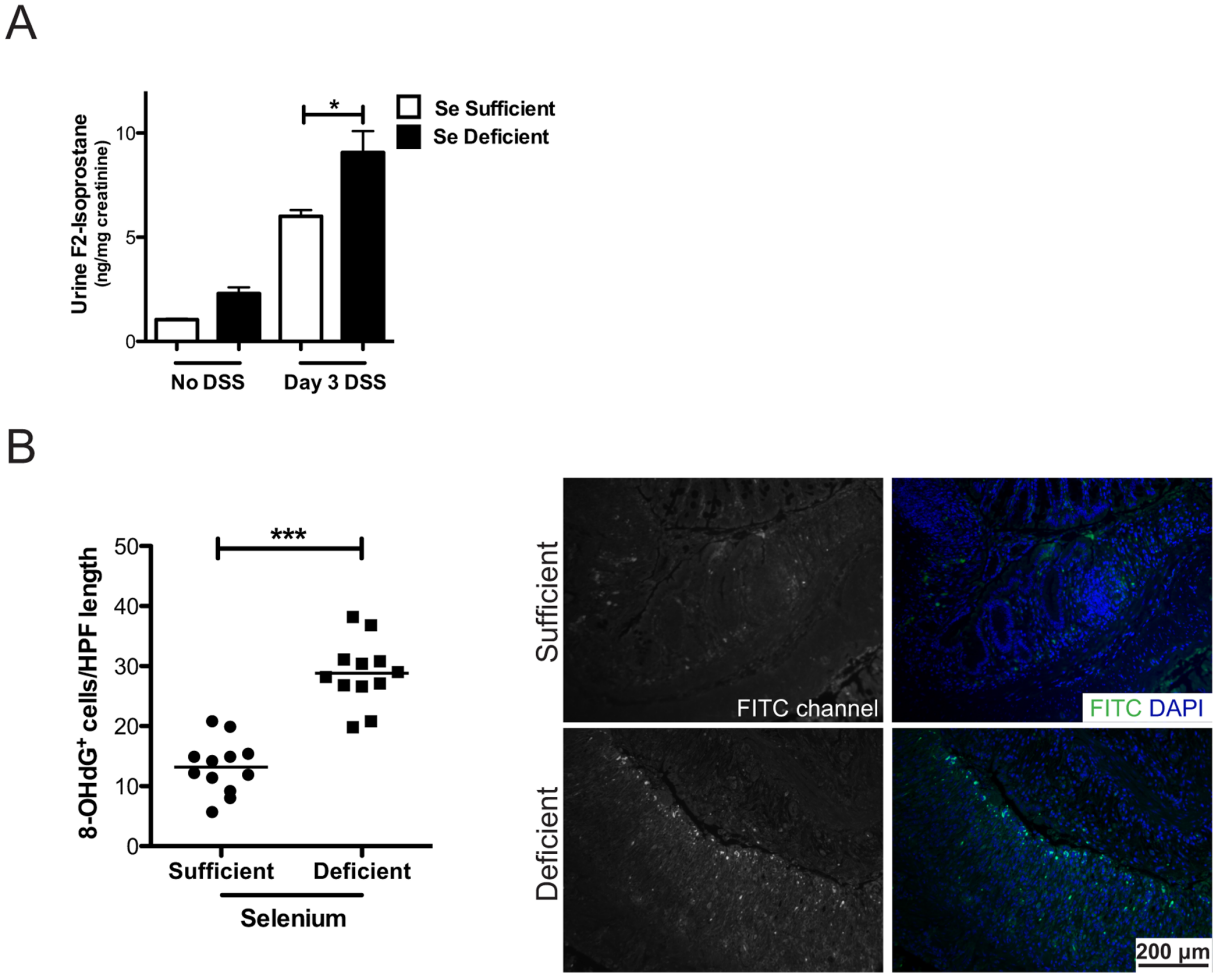
Selenium deficiency induces oxidative stress as measured by F_2_-isoprostanes and DNA damage. A. Selenium-sufficient (n = 3, white bars) and -deficient (n = 3, black bars) mice were given 3% DSS *ad lib.* and maintained in metabolic cages. Urine was collected at days 0 and 3 of DSS administration and F_2_-isoprostanes were measured and normalized to mg of creatinine. *P = 0.05, 1-way ANOVA. B. 8-OHdG (FITC) staining was performed in Se-sufficient (n = 12) and -deficient (n = 12) mice post-DSS morbidity protocol and quantified based on number of positive cells per high-powered field (HPF, 40x) length (left). Representative staining is shown on the right. ***P<0.0001.

For the DSS morbidity experiments (results shown in Figures 1, 2, 3, 4), Se-sufficient (n = 15) and Se-deficient (n = 10) mice were treated with DSS (Figure 1A) until their weight loss reached 20% of their original weights, at which time they were sacrificed. Animals were weighed daily and stools were examined for consistency and the presence of blood. Stool consistency scores ranged from 0–2, with 0 = hard, normal stools, 1 = soft and spread, 2 = diarrhea-like. Presence of blood was scored with a 0 = no blood, 2 = blood present, 4 = copious amounts of blood. Values were added for each mouse.

**Figure 5:**
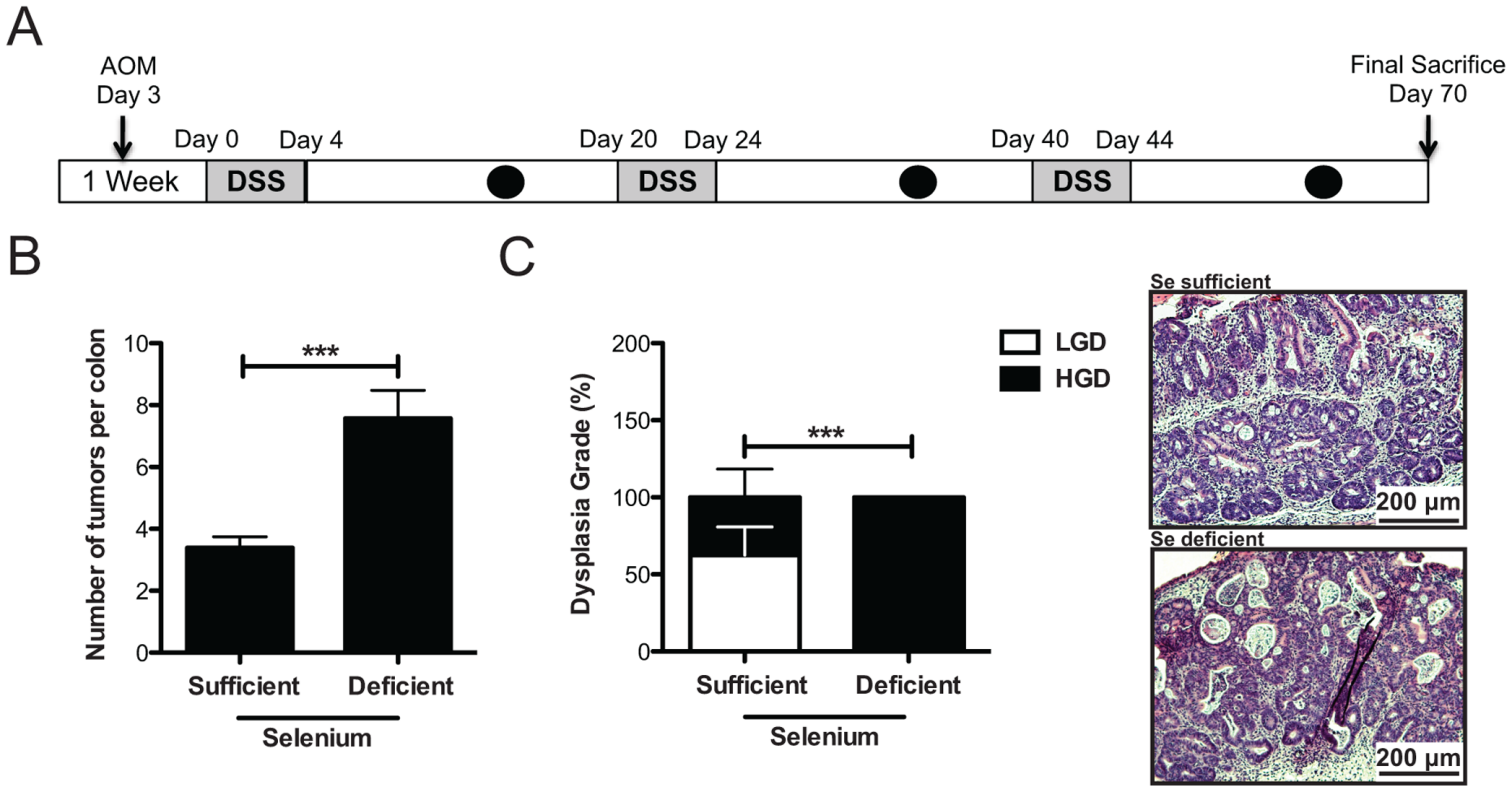
Selenium protects against tumor initiation and progression in inflammatory carcinogenesis. A. Schematic of the AOM/DSS CAC protocol. Mice are injected with AOM 4 days prior to beginning the first of three 4-day cycles of DSS. There are 16 days between each DSS cycle and mice are sacrificed 26 days after their last cycle of DSS. Black circles indicate periods at which mice are endoscopically analyzed. B. Selenium sufficient (n = 18) and deficient (n = 20) mice were subjected to the AOM/DSS protocol and tumor number was analyzed. ***P = 0.0002. C. Tumors were analyzed (by pathologist M.K. Washington) for dysplasia grade (LGD = low-grade dysplasia, HGD = high-grade dysplasia) and quantified based on percent of tumors demonstrating each grade (left, HGD ***P<0.0001, Fisher’s exact test). Representative H&E stained colons are displayed on the right.

**Figure 6:**
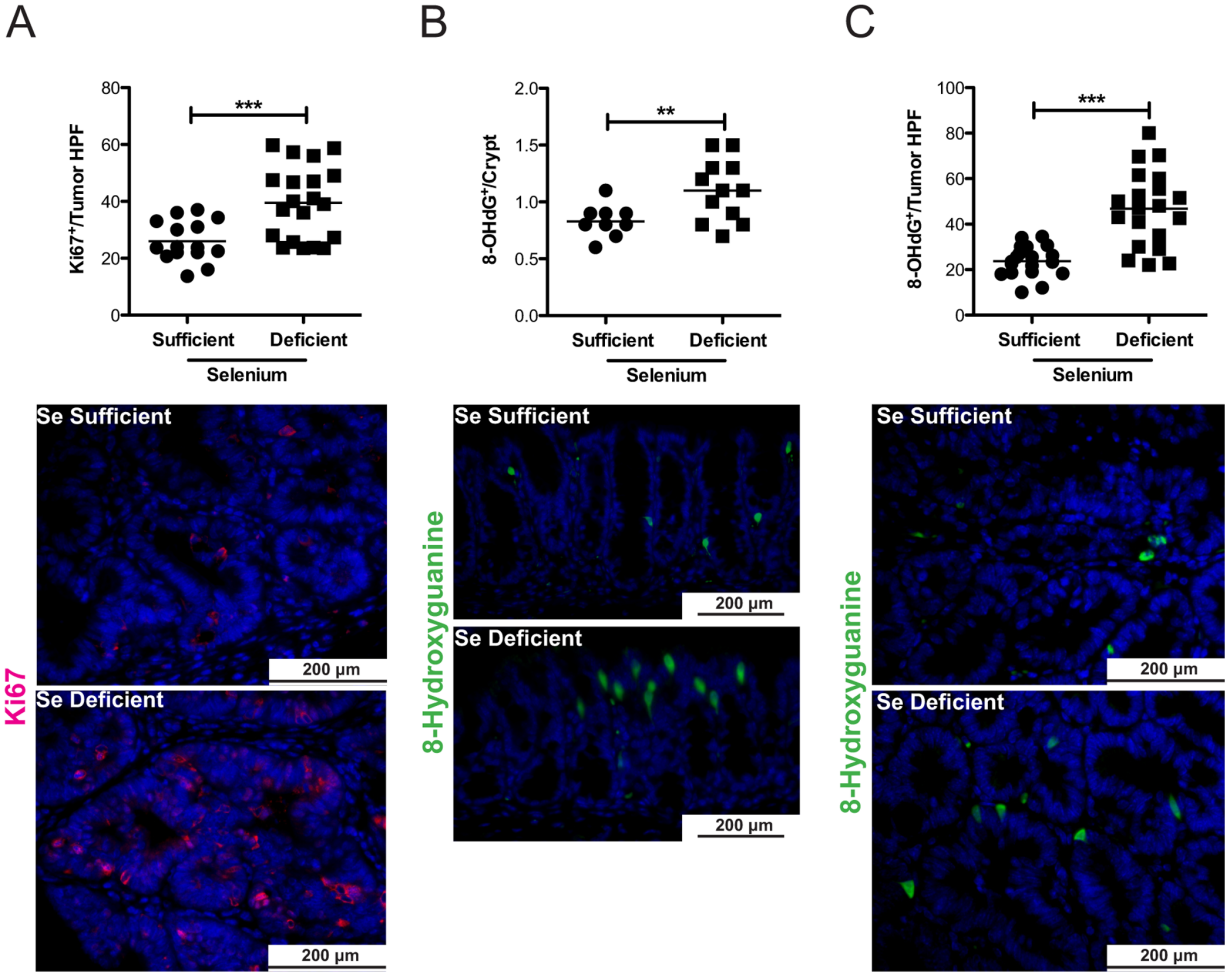
Selenium deficient colons demonstrate increased intratumoral proliferation and DNA damage in response to inflammatory carcinogenesis. Swiss-rolled colons of Se-sufficient and –deficient mice post-AOM/DSS protocol were analyzed for proliferation and DNA damage. A. Ki67 immunohistochemistry was conducted to identify actively proliferating cells. Intratumoral proliferation index calculated from number of Ki67-positive cells per HPF (top, Se-sufficient n = 15, Se-deficient n = 20, 40×). B. Immunofluorescence for 8-OHdG was conducted according to Materials and Methods. Staining is quantified per longitudinal crypt section in 20 crypts of each mouse (top) and representative images are shown (bottom). C. 8-OHdG staining is quantified per tumor HPF (top, Se-sufficient n = 19, Se-deficient n = 20, 40×) and representative images are shown (bottom). **P = 0.009, ***P<0.0001.

For the murine inflammatory carcinogenesis protocol (results shown in Figures 5–6), Se-sufficient (n = 18) or -deficient (n = 20) C57BL/6 WT mice were injected with 12.5 mg/kg AOM (Sigma-Aldrich) intraperitoneally as described in [33], [37], [38]. After a 3-day recovery period, the animals were started on the first of 3 cycles of 3% DSS ad libitum (see schematic in Figure 5A). Each cycle was 4 days in length and was separated by a 16-day recovery period. Eight days into each recovery period, murine endoscopy was performed to monitor colitis severity and tumor burden [39]. After the last cycle, animals were sacrificed following 26 days of recovery. Mice were anesthetized with isofluorane prior to exsanguination from the inferior vena cava. Blood was treated with 15 µl of a 1 mg/ml solution of disodium EDTA to prevent coagulation and plasma was separated by centrifugation for 2 minutes at 12,000×g. The colon was removed, flushed with PBS, and opened longitudinally and gross tumor counts were conducted using a dissecting microscope at 15× magnification and size measurements obtained using digital calipers. Microscopic analysis was conducted by an experienced gastrointestinal pathologist (M.K. Washington) for dysplasia on H&E stained “Swiss rolled” colons (processed by the Vanderbilt Translational Pathology Shared Resource core). Low-grade dysplasia (LGD) refers to preinvasive pedunculated, sessile, or flat plaque-like lesions with simple glandular outlines, without loss of cellularity polarity. High-grade dysplasia (HGD) are adenomas with architectural complexity, increased cytologic atypia, and loss of tumor cell polarity [40], [41]. Sections of tumor and normal adjacent colon were collected and stored in RNAlater (Qiagen, Valencia, Santa Clarita, California, USA) and a section of the most distal colon above the anus was collected and flash frozen in liquid nitrogen for protein analysis and/or determination of Se content. The remainder of the colon was “Swiss Rolled”, formalin fixed overnight, and sectioned for histological analysis.

### Colonic Selenium Measurements

The determination of colonic Se was carried out using a modification of the fluorometric assay of Koh and Benson [42] and Sheehan and Gao [43]. Briefly, tissue was digested in nitric and perchloric acids and Se was complexed with diaminonaphthalene. Selenium-diaminonaphthalene was extracted into cyclohexane and fluorescence was measured in a Perkin-Elmer LS 55 fluorometer.

### Plasma Glutathione Peroxidase Activity Assay

Plasma collected from mice was subjected to a glutathione peroxidase (Gpx) activity assay using a modified protocol [44] based on the method of Paglia and Valentine [45]. In short, 800 µl of reaction cocktail (50mmol/L potassium phosphate, pH 7.0, 1mmol/L EDTA, 1mmol/L NaN_3_, 0.2mmol/L glutathione) was mixed with 10 µl plasma and 90 µl water in a clear plastic cuvette and incubated at room temperature for 5 minutes. Next, 100 µl of H_2_O_2_ substrate (0.25mmol/L H_2_O_2_ in H_2_O) was added to the reaction mixture to initiate the reaction. Gpx activity was assessed by measuring the rate of change of A_340_ during the linear phase of the reaction. The rate of change was then converted to the number of µmoles of NADPH oxidized/minute using the extinction coefficient of 6.2×10^3^ L mol^−1^ cm^−1^ for NADPH at 340 nm. Blank reactions with distilled water were subtracted from each assay.

### F_2_-Isoprostane Analysis

DSS-treated mice (WT C57BL/6 fed Se-sufficient diets n = 3, WT C57BL/6 fed Se-deficient diets n = 3, 3% DSS ad libitum for 3 days) were housed in metabolic cages and urine was collected for 12 hours on days 0 and 3 of DSS administration. Urinary F_2_-isoprostanes were measured by stable isotope dilution gas chromatography mass spectrometry using [2H4]15-F2t-isoprostane as internal standard as previously described [46]. Values were normalized to mg creatinine in urine.

### Histological Injury Analysis

A colitis damage score was generated from digital images of H&E stained sections, by first measuring the distance around the intact muscularis using AVP Universal Desktop Ruler software (as performed by [47]). The areas of ulceration were then measured and given scores of 1, 2, 3, or 4, where a score of 1 represents injury involving the lower 1/3 of the crypt, 2 equaling injury to the lower 2/3^rds^ of the crypt, 3 equaling total crypt loss however the epithelial lining remains intact and 4 equaling total crypt and complete epithelial loss. The ulceration score was then multiplied by the length of the involved colon, summed, and divided by the total colon length to give a measure of ulceration severity/colon length. Measurements were performed in a blinded fashion in all colons from mice subjected to the DSS morbidity protocol.

### Cytokine Array Analysis

RNA from Se-sufficient or -deficient DSS morbidity colons (collected on day nine of DSS treatment) was isolated using the RNeasy Mini Kit (Qiagen, Valencia, Santa Clarita, California, USA). cDNA was synthesized using the iScript cDNA synthesis kit (Bio-rad, Hercules, California, USA) from 1 µg of total RNA. 1 µl of the 20 µl cDNA produced through the iScript reaction was used as a template in each subsequent PCR reaction. SYBR green qRT-PCR was performed using mouse cytokine array libraries I (Cat #: MCA-I) and II (Cat #: MCA-II) purchased from RealTimePrimers.com (Elkins Park, Pennsylvania, USA) according to manufacturer’s instructions. Individual cytokines were analyzed using the delta-delta Ct method and normalized to Hypoxanthine-Guanine Phosphoribosyltransferase *(Hprt).* Those showing significant differences were plotted using Graphpad Prism. In order to develop the heat map, data was incorporated into the free web-based PCR array analysis software provided by SABiosciences (Qiagen).

### Immunohistochemistry and Immunofluorescence Staining

Five-micrometer sections were cut, dewaxed, and hydrated. Antigen retrieval was conducted using Antigen Unmasking Reagent (Vector Laboratories, Inc., Burlingame, California, USA) according to manufacturer’s instructions. After blocking, primary antibody was added [α-Ki67 (NeoMarkers RB-1510-P, Fremont, California, USA), 1∶1,000; α-8-hydroxy-2′deoxyguanosine (8-OHdG, Abcam ab26842, Cambridge, Massachusetts, USA) 1∶50] and incubated overnight at 4°C. Isotype-matched antibodies were included as negative controls. Sections were then incubated for one hour at room temperature in secondary antibody (Ki67: Goat α-Rabbit-cy3, Invitrogen A10520; 8-OHdG: α-mouse-FITC, Sigma F-0257). Identification of apoptotic cells was conducted using the ApopTag Plus Peroxidase *In Situ* Apoptosis Kit (Chemicon, Temecula, California, USA) according to the manufacturer’s protocol. Control slides were obtained by omitting the terminal transferase (TnT) enzyme. For immunofluorescence staining of proliferation and DNA damage, slides were counterstained and mounted with ProLong Gold antifade including 4′,6-diamidino-2-phenylindole (DAPI, Invitrogen, Grand Island, New York, USA). DNA damage indices were generated by counting the number of positive cells per colon length within each high-powered field, with 5 high-powered fields counted per colon, starting at the most distal colon. Apoptosis, and proliferation indices were generated by counting either the number of positive cells per high-powered field (HPF; 40x objective) within each tumor or the number of positive cells per longitudinal crypt section in 20 crypts per mouse by a blinded observer. The average score was then calculated for each Swiss-rolled colon.

### Statistical Methods

Differences between Se-deficient and -sufficient mice were determined utilizing Graphpad Prism and analysis with the Student’s t-test. The survival curve was analyzed using the Log-rank (Mantel-Cox) test. Differences in stool scores and percentage weight loss were determined using two-way ANOVA for repeated measurements over time. Urine F_2_-isoprostane analysis of all four groups was analyzed using one-way ANOVA with a Newman-Keuls post-test to determine variances between columns. Finally, statistical analysis of high-grade dysplasia was performed using the Fisher’s Exact test.

## Results

### Dietary Selenium Maintains Epithelial Integrity

Se, through selenoproteins, has antioxidant roles, influences immune activity, and has been inversely correlated with IBD [4], [48] and cancer risk [1]. Because oxidative stress compromises mucosal integrity [49], and Se deficiency has been shown to increase oxidative stress [50], we determined whether dietary Se deficiency influences the severity of colitis following treatment with 3% DSS *ad lib* (Figure 1A). Animals were fed either Se-sufficient or -deficient diets for 12 weeks, an extended period of time sufficient to ensure Se deficiency which we confirmed via measurement of colonic mucosal Se levels at the end of the DSS experiment (333.1±16.5 vs 60.6±4.1 ng/g, P<0.0001, Figure 1B) and plasma glutathione peroxidase (Gpx) activity (215.4±31.6 vs 18.9±27.8 nmol NADPH/ml.min, *P*<0.0001, Figure 1C). In this experiment, mice were maintained on 3% DSS until their weight reached 80% of their original weights (survival endpoint). Se deficient mice median survival was 9 days in comparison to Se sufficient whose median survival was 12 days (P = 0.003, Figure 2A). Additionally, Se deficient mice had worsened diarrhea and increased blood in stools (stool scores at day 9∶2.7±0.7 vs 5.7±0.3, *P* = 0.02, Figure 2B), had accelerated weight loss (84.3±1.2 vs 77.4±1.2% of starting body weight at day 9, *P* = 0.01, Figure 2C), and had more severe colitis/epithelial injury (2.1±0.2 vs 2.9±0.1, *P* = 0.006, Figure 2D). These results indicate that Se is protective against DSS-induced colonic injury.

### Pro-tumorigenic Microenvironment is Promoted by Selenium Deficiency

Inflammation, a component of the epithelial and tumor microenvironment, is a contributing factor that predisposes to malignancy and, as we had observed increased injury in response to DSS administration in Se-deficient mice, we wanted to determine whether chemokine and cytokine signaling was aberrantly regulated in Se-deficient mice in response to injury. Therefore, we analyzed Se-sufficient and -deficient colons for cytokine mRNA expression using a mouse cytokine array. Because the RNA isolated for analysis was isolated from whole colon, the source of these cytokines and chemokines would include epithelial and stromal components. This analysis yielded alterations in several chemokines and cytokines that are displayed as a heat map for all genes tested (Figure S1A and B). Significantly altered cytokines were grouped according to function using PANTHER Pathway Analysis software [51]. Transforming growth factor beta (TGF-β) signaling was altered with increased *Tgf-β1* and -*β2* transcripts and decreased growth differentiation factor-1 (Gdf1) and bone morphogenetic protein 8b (Bmp8b) (Figure 3A). Additionally, the EGF pathway was altered (Figure 3B), as were components of developmental programs (Figure 3C). Levels of thyroid peroxidase (Tpo), PANTHER classified as an antioxidant, was increased (0.01±0.003 vs 9.1±6.3 fold change, P = 0.04, Figure 3D), consistent with a decrease in Se leading to aberrant regulation of thyroid hormone production [52]. Supporting immune system process activation there was increased chemokine (C-X-C motif) ligand 13 (Cxcl13), interferon kappa (Ifnk), cardiotrophin 1 (Ctf1), chemokine (C-C motif) ligand 27 (Ccl27), chemokine (C-C motif) ligand 20 (Ccl20), interleukin 17c (Il-17c), and interleukin 23a (Il-23a) messenger RNA in Se-deficient states (Figure 3E). Collectively, multiple factors capable of promoting tumor development were upregulated after DSS treatment in a Se-deficient state.

### Lipid Peroxidation, a Marker of Oxidative Stress, is Increased in the Urine of Selenium-deficient mice after DSS Induced Colitis

DSS administration in the drinking water causes colonic epithelial injury and a robust innate inflammatory response producing an environment enriched in reactive oxygen and nitrogen species [53]. Oxidative injury is quantifiable by measuring F_2_-isoprostanes (F_2_-IsoPs), one of the most reliable measures of in vivo lipid peroxidation, a marker of oxidative stress [46], [54]. We measured F_2_-IsoPs using stable isotope dilution gas chromatography mass spectrometry methodology. F_2_-IsoP levels were not different at baseline between Se-sufficient and -deficient mice (0 time point, Figure 4A). However, increased urine F_2_-IsoPs were observed in mice fed a Se-deficient diet when compared to mice on a Se-sufficient diet, after 3 days of 3% DSS (6.0±0.3 vs 9.1±1.0 ng/mg creatinine, P = 0.05, Figure 4A), indicating that Se protects from lipid peroxidation in this model. Therefore, F_2_-IsoPs effectively quantitate oxidative stress levels in the DSS model and Se-deficiency increases whole animal oxidative stress after DSS-induced injury.

### DNA Damage is Increased in Selenium-deficient mice in Response to DSS

As inflammatory injury and lipid peroxidation were upregulated in Se-deficient mice, and because Se has been linked with DNA damage repair [55], [56], we next determined whether DNA damage was increased in these mice. Colonic epithelial 8-OHdG staining, which identifies oxidative damage to DNA, was significantly increased in Se-deficient mice after DSS injury (13.2±1.3 vs 28.8±1.6 8-OHdG^+^ cells/HPF length, P<0.0001, Figure 4B). Thus, Se-deficiency exacerbated colitis and was associated with increased pro-inflammatory and pro-tumorigenic cytokines and increased DNA damage. These changes collectively produce a microenvironment primed for tumorigenesis.

### Dietary Selenium Protects from Inflammatory Carcinogenesis

Because Se deficiency promoted an activated pro-tumorigenic microenvironment and DNA damage after DSS treatment, we next determined if Se status modified inflammatory carcinogenesis. AOM is a procarcinogen that is metabolically activated to a potent alkylating agent forming O^6^-methyl-guanine [57]. Its oncogenic potential is markedly augmented in the setting of chronic inflammation, such as that induced by repeated cycles of DSS treatment [58], [59].

To determine if dietary Se modifies CAC, we modeled inflammatory carcinogenesis in Se-deficient or –sufficient C57BL/6 mice using AOM/DSS (Figure 5A). There were no significant changes in weight loss or DSS consumption during the course of this experiment (Figure S2D and E). At necropsy, Se-deficient mice demonstrated increased tumor multiplicity (3.4±0.4 vs 7.6±0.9 tumors/mouse, P = 0.0002, Figure 5B) as well as a higher degree of dysplasia (P<0.0001, Figure 5C) compared to Se-sufficient mice. Consistent with the morbidity studies (Figure 2), there was an increase in morbidity in Se-deficient mice in response to the AOM/DSS protocol (Figure S2A), though this difference was not statistically significant. We did not detect alterations in colon weight or length, however, suggesting that Se-deficient mice did not sustain chronic inflammatory changes in response to the AOM/DSS protocol (Figure S2B and C), at least when evaluated after the 26-day recovery period. Taken together, these data suggest that Se serves as a tumor suppressor that exerts its influence at the levels of tumor initiation and progression.

### Selenium Deficiency Results in Increased Intratumoral Proliferation and a General Increase in DNA Damage

Several selenoproteins have been identified as direct WNT targets [60] and Se deficiency activates WNT signaling [18], implicating Se deficiency in a proliferation pathway that is aberrantly regulated in colorectal cancer. Furthermore, mitochondrial selenoproteins have been implicated in the regulation of mitochondrial-dependent cell death [19]. As we noted an increase in tumor number in Se-deficient mice, we determined whether cellular proliferation or apoptosis indices were altered. Proliferation, as measured by Ki67 staining, was increased in the tumors of Se-deficient mice (26.0±1.9 vs 39.5±2.9 Ki67^+^ cells/tumor HPF, P = 0.0008, Figure 6A), but not within colonic crypts (Figure S3A). Apoptosis was quantified by TUNEL staining and no difference was seen between the two groups (Figure S3B and C). Interestingly, an increase in intratumoral proliferation is generally associated with an increase in tumor size and not number. For this reason, and due to the fact that DSS results in DNA damage [33], [61] which can lead to changes in the initiation frequency, we stained crypts and tumors of Se-sufficient and -deficient mice with 8-OHdG. Consistent with what was observed in the DSS morbidity study, Se deficiency led to a significant increase in DNA damage within crypts (0.83±0.04 vs 1.1±0.08 8-OHdG^+^/crypt, P = 0.009, Figure 6B), as well as tumors (23.7±1.5 vs 46.8±3.7 8-OHdG^+^/tumor HPF, P<0.0001, Figure 6C). Indicating augmented DNA damage, potentially contributing to the increased tumorigenesis observed in Se-deficient mice.

## Discussion

The essential trace element Se may be useful for the prevention and/or abrogation of several diseases related to oxidative stress including neurodegenerative, cardiovascular diseases [62]. In these diseases, Se deficiency is uncommon and thus therapeutic approaches targeting Se would require supranutritional amounts of Se for treatment. This point is important because harmful side-effects of supranutritional Se have been identified including increased risk for diabetes [63]. In contrast, IBD is a disease characterized by chronic inflammation, high oxidative stress and Se deficiency making it a potential target for Se supplementation, with the goal being to restore Se nutritional status to the normal range. In support of this concept, our results demonstrate that, in fact, Se sufficiency protects against DSS-induced injury when compared to Se deficiency as morbidity, weight loss, and mucosal injury in response to DSS were all significantly increased in mice fed a Se-deficient diet. Moreover, Se may protect against mucosal injury by decreasing expression of pro-inflammatory cytokines; as such we detected increased mRNA expression of the B cell chemoattractant chemokine (C-X-C motif) ligand 13 (Cxcl13), the T cell inflammation associated Chemokine (C-C motif) ligand 27 (Ccl27), the lymphocyte chemoattractant Chemokine (C-C motif) ligand 20 (Ccl20), as well as interleukin (Il)-17c which can promote Th17 cell responses [64]. Of particular interest is the upregulation of Il-23a, as Il-23 has been shown to be a pro-inflammatory factor and target protein for inhibitory therapy in Crohn’s disease, since it has major effects on Th17 cell differentiation [65]. These data suggest that restoration of normal Se levels may be a therapeutic option in IBD.

DSS-induced colonic injury, like ulcerative colitis, results in increased oxidative stress, which is at least partly attributable to immune cell infiltration and the release of inflammatory mediators [66], [67]. One method to quantify oxidative injury is to assess lipid peroxidation via measurement of F_2_-isoprostanes (F_2_-IsoPs), non-enzymatic derivatives of arachidonic acid [46]. It is important to note that, although lipid peroxidation is an indirect measure of oxidative stress, the Biomarkers of Oxidative Stress Study (BOSS) identified F_2_-IsoPs as the most reliable index of in vivo oxidative stress when compared against other known biomarkers [54]. Other labs have demonstrated increased F_2_-IsoPs in response to DSS using the Cayman assay [68], [69]. We utilized stable isotope dilution gas chromatography mass spectrometry analysis of F_2_-IsoPs to demonstrate that Se deficiency further augments the effects of DSS, indicating that DSS-induced lipid peroxidation, an indicator of oxidative injury, is increased in response to Se deficiency upon DSS administration and implicating Se in protection from lipid peroxidation in the DSS model.

Chronic inflammation and cyclic epithelial injury characteristic of IBD results in increased cancer risk, although the magnitude of this risk is unclear [35]. In our studies we found that Se deficiency leads to increased colonic injury and inflammation. Additionally, several TGF-β signaling components were aberrantly expressed in Se-deficient mice after DSS treatment. Transcripts for bone morphogenetic protein 8b (Bmp8b), which important for primordial germ cell determination [70], and a second TGF-β family member, growth differentiation factor 1 (Gdf1), were downregulated in DSS-treated Se-deficient mice. Gdf1 is correspondingly underexpressed in salivary gland adenoid cystic carcinoma [71] suggesting a potential role as a tumor suppressor. Furthermore, Tgf-β1 gene expression is upregulated in Se-deficient mice and has been implicated in the pathogenesis of colorectal neoplasia and several polymorphisms have been identified in colorectal adenoma which lead to Tgf-β1 overexpression [72], [73]. Tgf-β2, also identified as overexpressed in Se-deficient mice, has been linked with tumor promotion as it is upregulated in colorectal, breast, pancreatic, and brain cancer [74].

Transcripts of the EGF pathway are also upregulated in Se-deficient mice with pathway members including epidermal growth factor (Egf), insulin-like growth factor 1 (Igf1) and neuregulin 4 (Nrg4) all demonstrating increased expression in Se-deficient compared to -sufficient mice subjected to the DSS morbidity protocol. EGF is upregulated nearly 3-fold in colorectal carcinoma [75] and the EGF signaling pathway is intimately linked to promotion of solid tumor growth [76]. These data coupled with the fact that we also see increased expression of the ephrins Efnb2 and Efnb3, which are known to contribute to tumor promotion as well as progression [77], suggest that the signaling cascades and inflammation resulting from Se deficiency after DSS injury may result in increased cancer risk.

Beyond activation of oncogenic signaling pathways, Se deficiency may promote tumorigenesis via affecting genomic integrity [55]. Supplementation with moderate levels of Se compounds both in vivo and in vitro is thought to protect against the formation of DNA adducts and maintain chromosomal stability and telomere length and function [56]. In support of this we found that Se deficiency did, in fact, increase DNA adduct formation, a marker of DNA damage, after DSS-induced colonic stress, suggesting another mechanism by which Se might inhibit tumorigenesis in CAC.

Two prior studies report on the effects of reduced dietary Se in inflammatory colon tumorigenesis. Both have demonstrated a subtle, non-significant, increase in tumorigenesis with decreased dietary selenium [31], [78]. There are several potential explanations for why we observed a significant increase in tumor burden in our studies; 1) Differences in model selection, 2) strain of mice studied, and 3) degree and magnitude of Se deficiency. Our mice were fed Se-deficient diets (less than 0.01 mg Se/kg) for 12 weeks prior to the initiation of our protocols. This extensive time-frame was selected because our group has previously determined that this will ensure Se deficiency [79]. In our current studies we confirmed Se deficiency by measuring plasma Gpx activity and colonic Se levels. In contrast, the prior studies used low Se diets (0.02 and 0.086 mg Se/kg, respectively) for shorter time periods before protocol initiation. Our AOM/DSS protocol employs a single injection of AOM followed by cyclical administration of 3% DSS. Using this protocol, we achieved a tumor penetrance of 100% revealing a significant role of Se deficiency in inflammatory carcinogenesis. Furthermore, our data suggest that Se may also protect from tumor progression as more advanced lesions occurred in the setting of Se deficiency.

There is considerable epidemiological evidence indicating that Se levels and cancer are inversely correlated [20], [22]–[24]. Dietary intervention studies targeting Se have been inconsistent, however. Our findings suggest that Se supplementation, as a therapeutic intervention, should be targeted to patient populations, or individual patients, with demonstrable selenium deficiency. Importantly, our data indicate that patients with IBD, a disease in which Se deficiency occurs, might benefit from Se supplementation to prevent CAC. Beyond IBD and CAC, there needs to be a concerted effort towards fully understanding the molecular basis for how Se protects from tumorigenesis, in what cancers Se is important, at what dietary levels Se is most beneficial, and in which patient populations selenium supplementation might have a beneficial impact.

## Supporting Information

Figure S1 **Cytokines are aberrantly regulated in seleniumdeficient mice subjected to DSS.** A. Heat map of mouse cytokine library I and B. mouse cytokine library II gene expression in Se-deficient (n = 4) versus -sufficient (control, n = 4) colons post-DSS morbidity (left) and a chart of represented cytokines (right). Green = underexpression, Red = overexpression where intensity of color indicates increasing distance from 0; Grey = qRT-PCR yields were undetectable.
(TIF)

Figure S2 **Selenium levels do not significantly impact survival, colon weight, colon length, or mouse weight in response to AOM/DSS.** A. Survival curve of Se-deficient and -sufficient mice in response to the AOM/DSS protocol. B. Colon weight (g) and C. colon length (mm) post-AOM/DSS protocol. D. Mouse weight (g) throughout the AOM/DSS protocol measured on days 0, 1, 3, 4, and 7 of DSS administration during each DSS cycle. E. DSS consumption/mouse during the four days of DSS administration.
(TIF)

Figure S3 **Crypt proliferation and crypt and tumor apoptosis are unaltered in dietary models in response to AOM/DSS.** A. Quantification of Ki67 staining within crypts in non-tumor bearing areas of Se-sufficient and -deficient mice. 20 crypts were counted for each mouse. B. Crypt and C. intratumoral TUNEL counts in Se-sufficient and -deficient mice.
(TIF)
