# Supplementary figures and images for: Dietary Selenium Deficiency Exacerbates DSS-Induced Epithelial Injury and AOM/DSS-Induced Tumorigenesis
Source: PLoS One. 2013 Jul 4;8(7):e67845. doi: 10.1371/journal.pone.0067845 (PMC3701622; doi:10.1371/journal.pone.0067845)

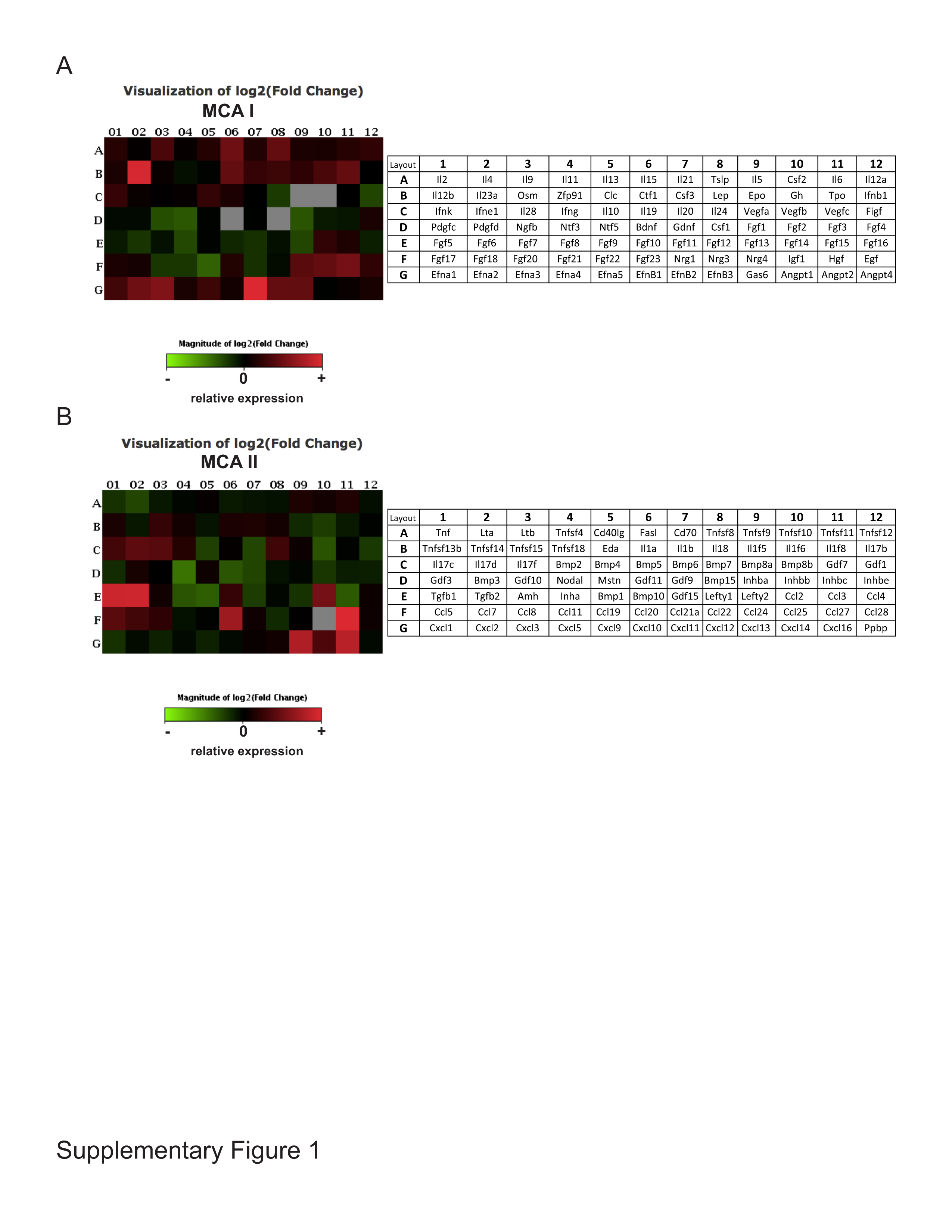

Supplement: Figure S1 — Cytokines are aberrantly regulated in seleniumdeficient mice subjected to DSS. A. Heat map of mouse cytokine library I and B. mouse cytokine library II gene expression in Se-deficient (n = 4) versus -sufficient (control, n = 4) colons post-DSS morbidity (left) and a chart of represented cytokines (right). Green = underexpression, Red = overexpression where intensity of color indicates increasing distance from 0; Grey = qRT-PCR yields were undetectable. (TIF) [file pone.0067845.s001.tif]

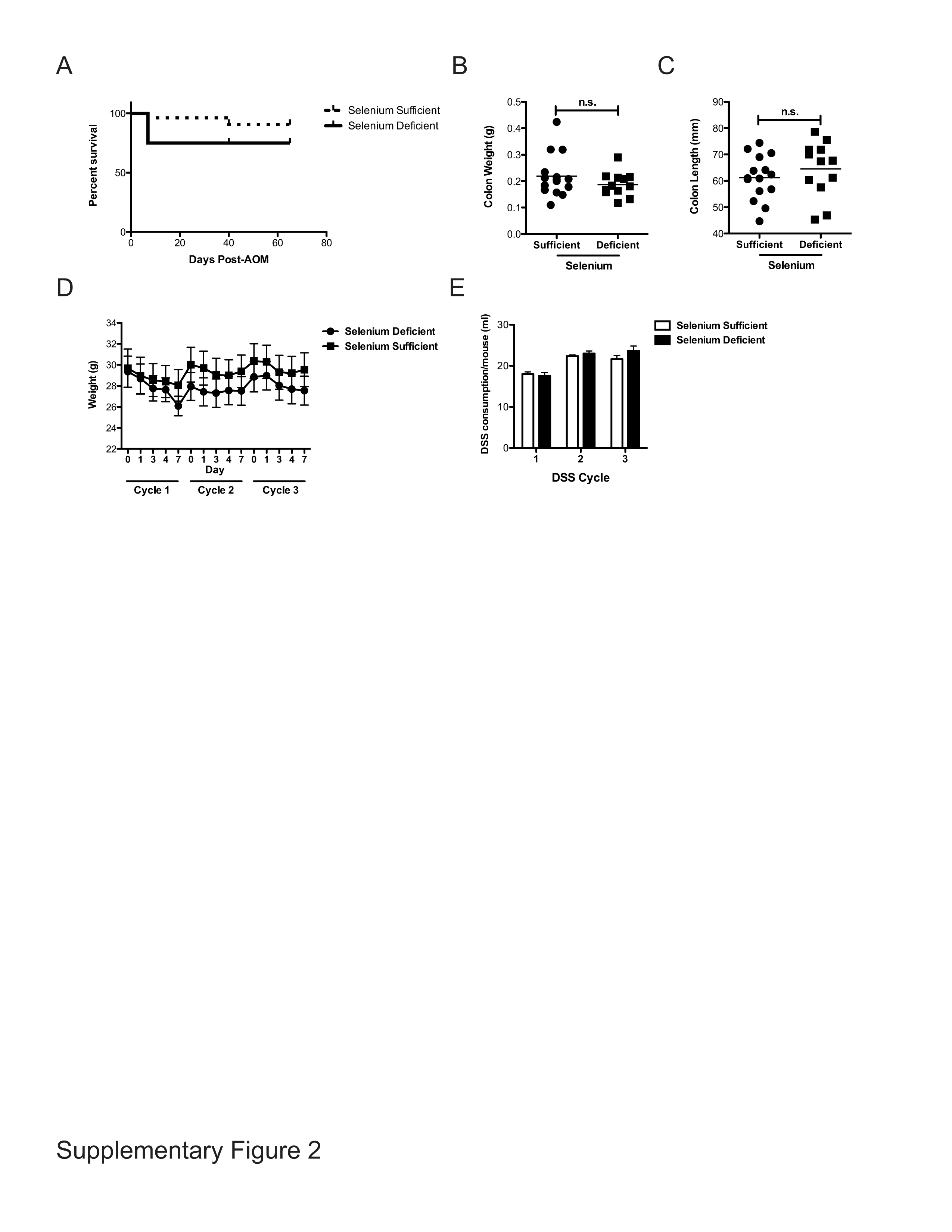

Supplement: Figure S2 — Selenium levels do not significantly impact survival, colon weight, colon length, or mouse weight in response to AOM/DSS. A. Survival curve of Se-deficient and -sufficient mice in response to the AOM/DSS protocol. B. Colon weight (g) and C. colon length (mm) post-AOM/DSS protocol. D. Mouse weight (g) throughout the AOM/DSS protocol measured on days 0, 1, 3, 4, and 7 of DSS administration during each DSS cycle. E. DSS consumption/mouse during the four days of DSS administration. (TIF) [file pone.0067845.s002.tif]

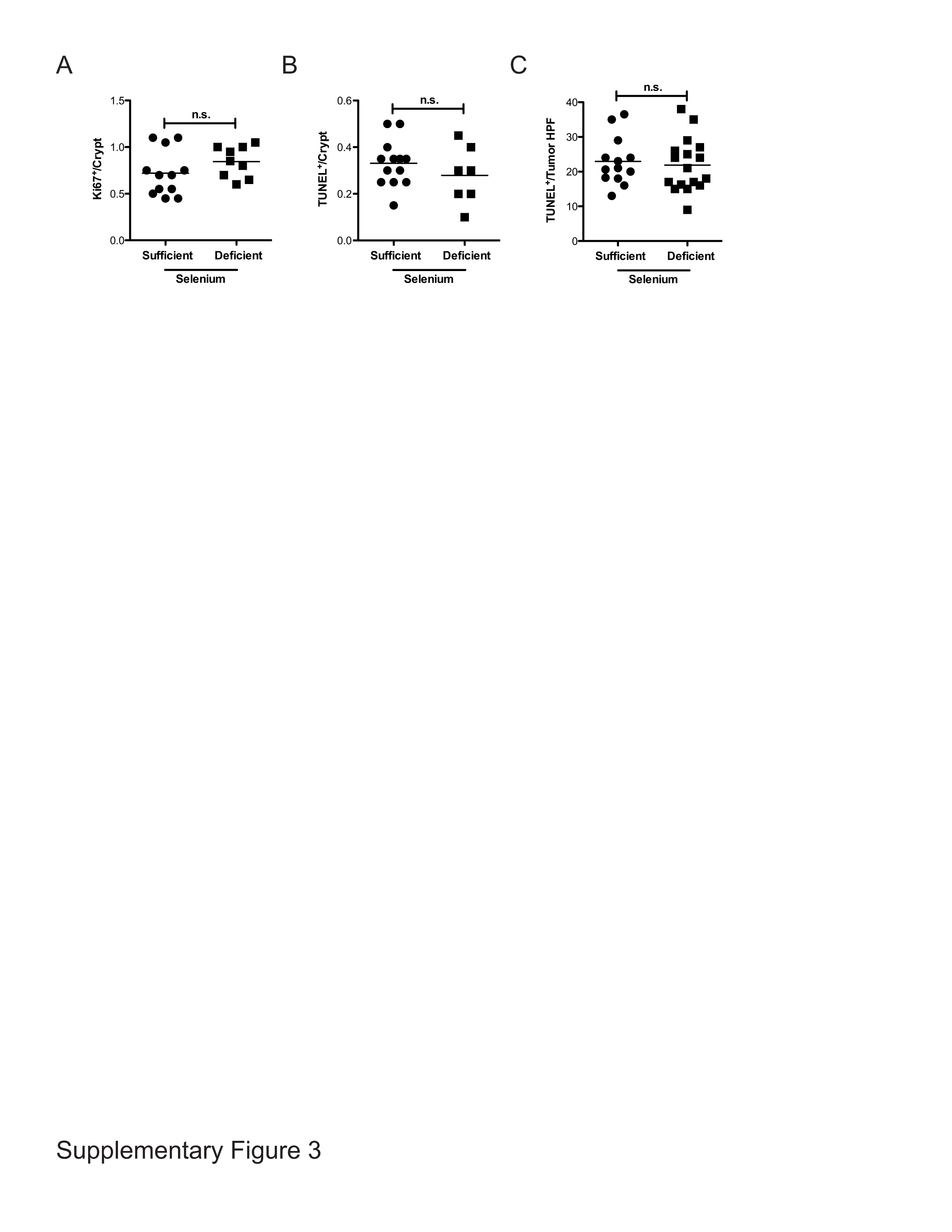

Supplement: Figure S3 — Crypt proliferation and crypt and tumor apoptosis are unaltered in dietary models in response to AOM/DSS. A. Quantification of Ki67 staining within crypts in non-tumor bearing areas of Se-sufficient and -deficient mice. 20 crypts were counted for each mouse. B. Crypt and C. intratumoral TUNEL counts in Se-sufficient and -deficient mice. (TIF) [file pone.0067845.s003.tif]
